# A rare case of non-invasive ductal carcinoma of the breast coexisting with follicular lymphoma: A case report with a review of the literature

**DOI:** 10.3892/ol.2014.1885

**Published:** 2014-02-14

**Authors:** MIKAKO TAMAOKI, YOSHINORI NIO, KAZUHIKO TSUBOI, MARIKA NIO, MASASHI TAMAOKI, RIRUKE MARUYAMA

**Affiliations:** 1 Nio Breast Surgery Clinic, Kyoto, Japan; 2 Department of Pathology, Faculty of Medicine, Shimane University, Izumo, Japan

**Keywords:** breast cancer, ductal carcinoma *in situ*, follicular lymphoma, double malignancies

## Abstract

The double presentation of breast cancer and follicular lymphoma is extremely rare, and only six cases have previously been reported in the literature. In the present study, a case of synchronous ductal carcinoma *in situ* (DCIS) of the breast and follicular lymphoma is reported. During an annual breast screening procedure, a 49-year-old female presented with a hard induration under the nipple of the right breast and swelling of a soft lymph node (LN) in the right axilla. Mammography and ultrasonography revealed two lesions in the right breast: One was a tumor with microcalcification, 1.0 cm in diameter, and the other was a large, crude calcification, 2.5 cm in diameter. In addition, computed tomography and positron emission tomography revealed swellings of the bilateral axillary (Ax) LN and intra-abdominal para-aortic LN. The patient underwent excisions of the large calcified mass, a micro-calcified tumor and the right AxLN. The pathological and immunohistochemical studies revealed fat necrosis and DCIS of the breast, which was positive for the estrogen receptor and the progesterone receptor, while human epidermal growth factor receptor II protein expression was evaluated as 2+ and stage was classified as pTis pN0 M0, stage 0. Furthermore, the Ax node was diagnosed as follicular lymphoma, which was positive for cluster of differentiation (CD)20, CD79a, CD10 and B-cell lymphoma (Bcl)-2 protein, but negative for Bcl-6 protein. The clinical stage was classified as stage III. The patient was administered chemotherapy followed by radiotherapy to the conserved breast. Two years have passed since the surgery, and the patient is disease-free.

## Introduction

The incidence of breast cancer (BC) is extremely high, and malignant lymphoma (ML) is a common malignant disease. It is also well known that BC is the most frequent secondary malignancy following treatment for Hodgkin’s lymphoma (HL), particularly in young females who receive radiotherapy for early-stage HL (1–3). By contrast, the incidence of ML, including HL and non-HL (NHL) as second malignancies following breast conserving surgery and radiotherapy (RT) for BC, is rare (4,5).

Follicular lymphoma (FL) is classified as an NHL, amongst which FL is categorized as a low-grade ML and grows slowly. The incidence of FL is 20–30% of all ML in Europe and the USA (6), but in Japan it is only 10–15%, although it is increasing (7–9). NHL is rarely observed in the synchronous and metachronous presentation with BC, and the double presentation of BC and FL is even rarer; previously, only six cases, including metachronous and synchronous double presentation, have been reported in the literature (10–15). In the present study, a case of synchronous ductal carcinoma *in situ* (DCIS) of the breast and FL is reported, with a review of the literature.

## Case report

The current study describes the case of a 49-year-old female who had previously undergone bilateral breast augmentation with autologous fatty tissue injection in her youth. Prior to the study, the patient attended yearly breast screening appointments. In the most recent breast screening, the patient exhibited no obvious complaints and a mammography (MMG) examination was performed. In previous MMG examinations, no abnormal findings had been identified. The patient had undergone an autologous fat-tissue transplantation 10 years earlier. On palpation, a hard induration was palpated under the nipple of the right breast, and swelling of a soft lymph node (LN) was also palpated in the right axilla.

**Figure 1:**
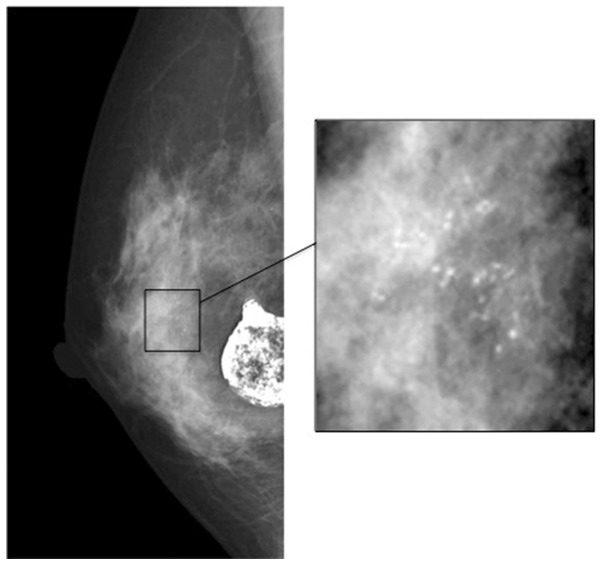
MMG revealing two lesions. As shown in the rectangle, one lesion was grouped as micro-calcifications in a ~1 cm^2^ area under the right nipple, which was classified as DCIS. Another was a large crude calcification of 2.5 cm in diameter, which was fat necrosis due to autologous transplantation of fatty tissue in the patient’s youth. DCIS, ductal carcinoma *in situ*; MMG, mammography.

**Figure 2:**
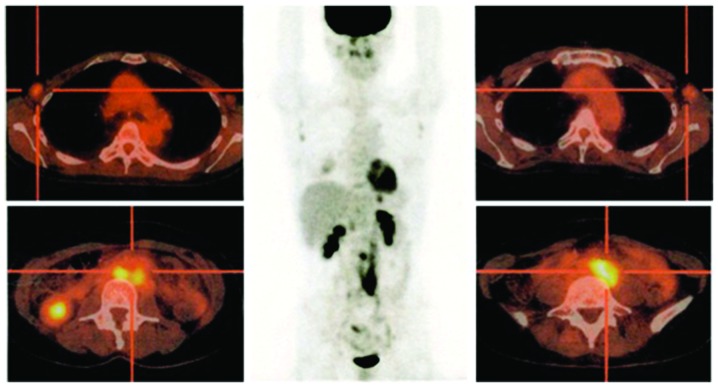
PET demonstrating an accumulation of ^18^F-fluorodeoxyglucose in the bilateral AxLNs (SUVmax, 2.8) and intra-abdominal para-aortic LNs (SUVmax, 5.8). PET, positron emission tomography; Ax, bilateral axillary; LN, lymph node; SUVmax, maximum standardized uptake value.

An MMG examination revealed two lesions: One consisted of a group of micro-calcifications in a ~1 cm^2^ area under the right nipple, and the other was a large crude calcification, 2.5 cm in diameter (Fig. 1). Ultrasonography examination revealed a low echoic lesion, including micro-calcifications and a large calcified mass. The aforementioned examinations indicated DCIS and fat necrosis following autologous fat-tissue transplantation. Computed tomography (CT) examination revealed a crude calcification and a lesion enhanced by a contrast drug. In addition, bilateral axillary (Ax) and intra-abdominal para-aortic LN swelling were revealed. Positron emission tomography (PET) also demonstrated accumulations of ^18^F-fluorodeoxyglucose in the bilateral AxLNs and intra-abdominal para-aortic LNs (Fig. 2). These findings indicated malignant lymphoma rather than metastasis from the breast DCIS.

**Figure 3:**
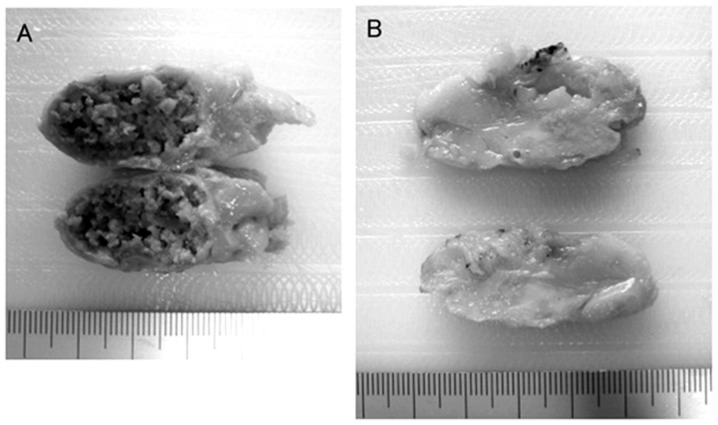
Macroscopic findings of surgical specimens. (A) A large calcified mass of fat necrosis. (B) A micro-calcified tumor of ductal carcinoma *in situ (*DCIS).

**Figure 4:**
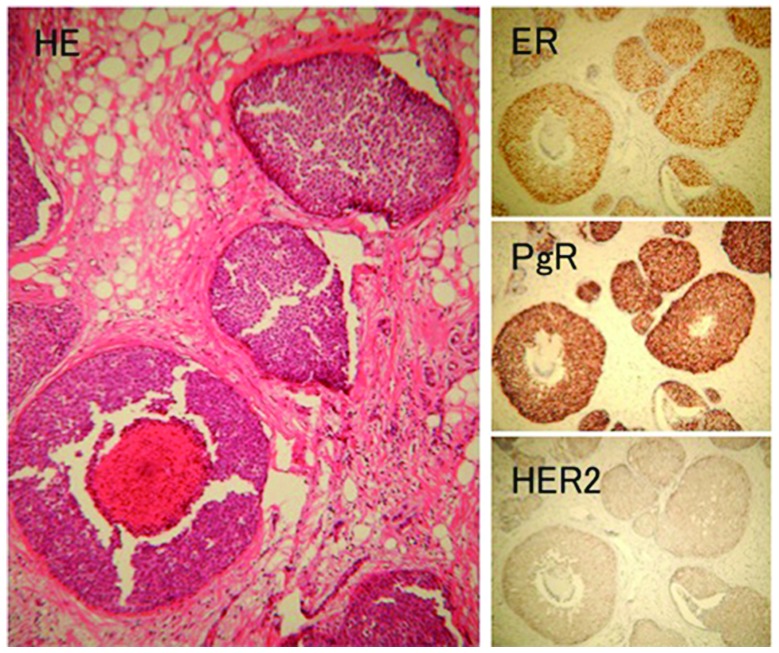
Pathology and immunohistochemistry of the ductal carcinoma *in situ* (DCIS). Magnification, ×100. DCIS was positive for estrogen receptor (ER) and progesterone receptor (PgR), and human epidermal growth factor receptor II (HER2) protein expression was evaluated as 2+.

**Figure 5:**
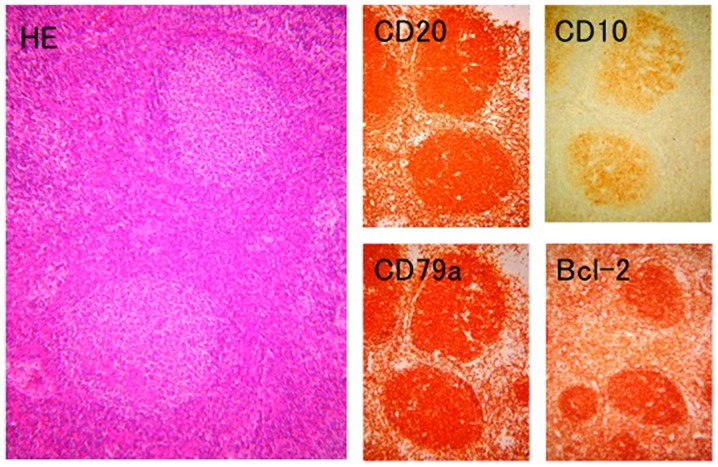
Pathology and immunohistochemistry of FL. Magnification, ×100. Tumor cells were positive for CD20, CD79a, CD10 and Bcl-2 protein. FL, follicular lymphoma; CD, cluster of differentiation; Bcl, B cell lymphoma.

The patient underwent excision of the large calcified mass (Fig. 3A), a micro-calcified tumor (Fig. 3B) and the right AxLN. The pathological diagnoses demonstrated that the large calcified mass was fat necrosis and the micro-calcified tumor was DCIS (Fig. 4). For immunohistochemical (IHC) examination, 4-μm sections of formalin-fixed, paraffin-embedded specimens were immunostained primarily according to the labeled polymer method using Dako EnVision™ kit (Dako, Carpinteria, CA, USA), according to the manufacturer’s instructions. The primary antibodies were purchased from Roche Diagnostics Japan (Tokyo, Japan) as follows: anti-estrogen receptor (ER) rabbit monoclonal antibody (mAb) (SP1), anti-progesterone receptor (PgR) rabbit mAb (1E2) and anti-human epidermal growth factor receptor II (HER2/new) rabbit mAb and from DakoCytomation (Glostrup, Denmark) as follows: anti-human cluster of differentiation (CD)20 mouse mAb, anti-CD79a mouse mAb, anti-CD10 mouse mAb, anti-B-cell lymphoma (Bcl)-2 mouse mAb and anti-Bcl-6 mouse mAb. IHC examinations revealed that the DCIS was positive for ER, PR and HER2 protein expression and was evaluated as 2+. The post-surgical stage classification was pTis pN0 M0, stage 0. The AxLN was diagnosed as FL (Fig. 5), as IHC examinations revealed that the tumor cells were positive for CD20, CD79a, CD10 and Bcl-2 protein (Fig. 5), but negative for Bcl-6 protein. The clinical stage was classified as stage III.

Treatment for FL was preferentially continued, as BC is a DCIS. The patient was administered combination chemotherapy with 600 mg rituximab, 1,100 mg cyclophosphamide, 2 mg vincristine and 80 mg prednisolone (R-CVP) at 3-week intervals for 6 cycles, and the clinical response was evaluated as a complete response. Subsequent to R-CVP therapy, the patient received radiotherapy (RT) to the conserved breast 25 times at 2.0 Gy. In total, RT was received 5 days a week for 5 weeks (total dose, 50 Gy). Subsequent to RT, the patient was administered a luteinizing hormone-releasing hormone agonist, leuprorelin acetate, at 3.75 mg at 4-week intervals. Two years have passed since the surgery, and the patient is disease-free. The patient provided written informed consent.

## Discussion

Synchronous or metachronous presentations of BC and FL are rare, and to the best of our knowledge, only six cases have previously been reported in the literature (10–14); the present study is the seventh case. Profiles of the seven cases are summarized in Table I. Of the seven cases, only one case was a metachronous presentation, and the FL occurred two and a half years after the BC. Six cases were synchronous presentations. The BCs of the seven cases included five invasive ductal carcinomas (IDC) and two DCISs; four cases had left-sided BCs and three had right-sided BCs. The surgeries included three mastectomies and four breast-conserving surgeries, and the stages were classified as stage 0 in two cases, stage I in three cases, stage IIA in one case and stage IIB in one case. ER was positive in all five cases that were fully described, and following the surgery, six cases were administered adjuvant therapies.

FL was classified as stage IA in two cases, stage III in three cases and unclear in two cases. The biopsy sites for pathological diagnosis included six AxLNs and one breast. Histological grades were described in five cases and all of them were classified as low grade or grade 1. Surface markers were studied in five cases and all of them were positive for CD20, CD10 and Bcl-2 protein. CD79a was positive in two reported cases and Bcl-6 protein was positive in the present case, but negative in another case reported. The treatment was described in five cases: The patient of the present case was administered R-CVP, while in the other studies, one patient received CVP, one received CB and dexamethasone, one received adriamycin and cyclophosphamide and the other patient received no treatment.

The double presentation of BC and ML is not so rare, however, the majority are cases of individuals, particularly young females, who exhibit BC as a secondary malignancy subsequent to RT or chemotherapy for HL (1–3). The double presentation of BC and NHL is rare and to the best of our knowledge, a total of 32 cases, including the present case, have been reported in the literature (15–29). Besides seven cases with FL, the double presentations of NHL and BC have accounted for 25 cases, including 22 synchronous and three metachronous presentations; the profiles are summarized in Table II. Among them, chronic lymphocytic leukemia/small lymphocytic lymphoma were most frequently observed in eight cases.

In the present literature review, in 25 of 32 cases (78%) of double presentation, NHLs were diagnosed by pathologically examining AxLNs. This indicated that the excisional biopsy of the AxLN is the most important factor for identifying ML presenting with BC. For IDC, there is no problem in terms of the diagnosis of ML, as a sentinel node biopsy (SNB) or Ax dissection are the standard procedures. On the other hand, for DCIS, the diagnosis of ML is not always easy, as SNB of an AxLN is not indicated as a standard procedure for DCIS. However, a previous meta-analysis of SNB in DCIS demonstrated that the estimate for the incidence of SN metastases in a patient with a pre-operative diagnosis of DCIS was 7.4% compared with 3.7% in patients with a definitive diagnosis of DCIS alone, which indicated that SNB should be considered in patients with a pre-operative diagnosis of DCIS (30). According to the present literature review, SNB may be indicated in cases of DCIS of the breast when the AxLNs are swelling. Furthermore, pre-operative PET/CT examination, if possible, may also be beneficial in detecting metastasis and in identifying other malignant diseases of the LN.

## Figures and Tables

**Table I:** BC coexisting with FL.

	Patient						
		
Characteristics	1	2	3	4	5	6	Present case
Age, years	51	61	50	58	52	74	47
Gender	Female	Female	Female	Female	Female	Female	Female
Syn/meta	Metachro	Synchro	Synchro	Synchro	Synchro	Synchro	Synchro
BC							
Side of Breast	R	L	L	L	R	L	R
Histology	IDC	IDC	IDC	DCIS	IDC	IDC	DCIS
Grade		2	1		2	2	
T	1	1	1	cis	3	1	cis
N	0	1	0	0	0	0	0
M	0	0	0	0	0	0	0
Stage	I	IIA	I	0	IIB	I	0
ER	(+)		(+)		(+)	(+)	(+)
PgR	(−)		(+)		(+)	(+)	(+)
HER2						(−)	2+
Surgery	MX	WLE	WLE	MX	MX	WLE	WLE
Adjuvant therapy	FT	RT+Chem	RT+TAM	None	TAM	RT+AI	LP
FL							
Biopsy site	L-Br	AxLN	AxLN	AxLN	AxLN	AxLN	AxLN
Grade	Low		1	Low		1	1
Stage		III	IIIA	IA	IA		III
CD20		(+)	(+)		(+)	(+)	(+)
CD23		(+)					
CD79a			(+)				(+)
CD10		(+)	(+)		(+)	(+)	(+)
Bcl-2		(+)	(+)		(+)	(+)	(+)
Bcl-6			(+)				(−)
Cyclin D1			(−)				
Therapy	CVP		CB+DM	None	AC		R-CVP
Reference	10	11	12	12	13	14	
Year	1989	2005	2006	2006	2010	2010	2011

BC, breast cancer; FL, follicular lymphoma; metachro, metachronous; synchro, synchronous; R, right; L, left; Br, breast; IDC, invasive ductal carcinomas; DCIS, ductal carcinoma *in situ*; T, tumor; N, node; M, metastasis; MX, masectomy; RT, radiotherapy; FT, futraful; Chem, chemotherapy; TAM, tamoxifen; AI, aromatase inhibitor; LP, leuprorelin acetate; Ax, axillary; LN, lymph node; CD, cluster of differentiation; Bcl, B-cell lymphoma; CVP, cyclophosphamide + vincristine + predonosolone; CB + DM, chlorambucil + dexamethasone; AC, adriamycin + cyclophosphamide; R-CVP, rituxan + CVP.

**Table II:** Double presentation of BC and NHL.

Case no.	Age, years	Gender	BC			NHL			Ref.	Year
	
			Side	Histol	Stage	Histol	Biopsy location	Stage		
Synchro										
1	66	F	R	IDC	2A	BL	AxLN		16	1990
2	77	F	L	IDC	1	SLL	AxLN		16	1990
3	77	F	R	ILC	1	LPL	AxLN	3B	17	1994
4	77	F	L	Paget+DCIS	0	BL	AxLN	1A	17	1994
5	83	M	L	IDC		LPL	AxLN	1A	17	1994
6	62	F	R	IDC	3A	SLL/CLL	AxLN		18	1997
7	62	F	L	IDC	1	DLBCL	R-Br		19	2002
8	67	F	L	IDC	1	MCL	AxLN	1	20	2003
9	79	F	L	IDC	2A	MZBL	AxLN		21	2004
10	53	F	L	IDC	2A	MALT	AxLN		22	2006
11	63	F	L	IDC	1	MCL	AxLN		12	2006
12	56	F	L	ILC	2A	MZBL	AxLN	4	23	2008
13	57	F	Bil	IDCx2	Both 1	MZBL	AxLN		24	2008
14	69	F	R	IDC	1	DLBCL	R-Br		25	2009
15	74	F	R	IDCx2	2B	CLL/SLL	AxLN	0	14	2010
16	54	F	L	IDC	2A	SLL	AxLN		14	2010
17	52	F	L	IDC		DLBCL	Nasopharynx		26	2011
18	87	F	n.d.	IDC		CLL/SLL	AxLN		27	2011
19	69	F	n.d.	DCIS		CLL/SLL	AxLN		27	2011
20	62	F	n.d.	IDC		CLL/SLL	AxLN		27	2011
21	58	F	n.d.	IDC		CLL/SLL	AxLN		27	2011
22	67	F	n.d.	IDC		CLL/SLL	AxLN		27	2011
Metachro										
1	53	F	n.d.	IDC	n.d.	LPL	Parotid gland	2A	28	1990
2	55	F	L	IDC	2B	AILT	Neck LN	2	29	2003
3	53	F	R	IDC	2B	LPL			15	2004

BC, breast cancer; NHL, non-Hodgkin’s lymphoma; R, right; L, left; Bil, bilateral; Histol, histology; Synchro, synchronous; metachro, metachronous; F, female, M, male; IDC, invasive ductal carcinoma; DCCIS, ductal carcinoma *in situ*; BL, B-cell lymphoma; SLL, small lymphocytic lymphoma; LPL, lymphoplasmacytic lymphoma; CLL, chronic lymphocytic leukemia; DLBCL, diffuse large B-cell lymphoma; MCL, mantle-cell lymphoma; MZBL, marginal zone B-cell lymphoma; MALT, B-cell lymphoma of mucosa-associated lymphoid tissue; AILT, angioimmunoblastic T-cell lymphoma; LN, lymph node; Ax, axillary; Br, breast; n.d., no description.

## Abbreviations

Ax: axillary
BC: breast cancer
CT: computed tomography
DCIS: ductal carcinoma *in situ*
ER: estrogen receptor
FL: follicular lymphoma
HL: Hodgkin’s lymphoma
IDC: invasive ductal carcinoma
LN: lymph node
ML: malignant lymphoma
MMG: mammography
NHL: non-Hodgkin lymphoma
RT: radiotherapy
SNB: sentinel node biopsy
